# Functional Variant in the *GCKR* Gene Affects Lactate Levels Differentially in the Fasting State and During Hyperglycemia

**DOI:** 10.1038/s41598-018-34501-9

**Published:** 2018-10-30

**Authors:** Maykel López Rodríguez, Lilian Fernandes Silva, Jagadish Vangipurapu, Shalem Modi, Johanna Kuusisto, Minna U. Kaikkonen, Markku Laakso

**Affiliations:** 1 Institute of Clinical Medicine, Internal Medicine, University of Eastern Finland, Kuopio, Finland; 2 Department of Medicine, Kuopio University Hospital, P.O. Box 100 FI 70029 KYS, Kuopio, Finland; 3 A.I. Virtanen Institute for Molecular Sciences, Department of Biotechnology and Molecular Medicine, University of Eastern Finland, Kuopio, Finland

## Abstract

The rs780094 single nucleotide polymorphism (SNP; C/T) of glucokinase regulatory protein gene (*GCKR*) is a regulatory genetic variant that has been associated with lactate levels in the fasting state. However, the association of this locus with lactate during hyperglycemia, and the mechanisms underlying these associations remain unknown. We investigated the association of rs780094 with lactate levels in a frequently sampled oral glucose tolerance test in humans and evaluated the effect of increasing *GCKR* expression on lactate production in liver cells. The C allele of rs780094 was associated with lower lactate levels in fasting but increased lactate level during hyperglycemia independently of insulin levels. Increased expression of GKRP induced higher lactate level in HepG2 cells and in human primary hepatocytes (HPH) upon glucose stimulation by increasing the amount of GCK. Glucagon induced the expression of *GCKR* in HepG2 and HPH cells. Our results suggest that the association of rs780094 with lactate levels may involve differential *GCKR* expression between the carriers of the C and T alleles.

## Introduction

Genome-wide association studies (GWAS) have identified over 100 gene variants associated with type 2 diabetes (T2D), including the Glucokinase Regulatory Protein gene (*GCKR*; MIM 613463)^1^. Glucokinase Regulatory Protein (GKRP; NP_001477), encoded by *GCKR*, is almost exclusively expressed in the liver, where it inhibits glucokinase (GCK) in the nucleus of the hepatocytes at low glucose concentrations^2^. An intronic variant rs780094 and a coding variant rs1260326 of *GCKR* are in high linkage disequilibrium (LD), and these variants have been found to be associated with glucose and lactate levels, and a wide range of other metabolic traits^3–9^, *de novo* lipogenesis in obese young individuals^10^, and the risk of non-alcoholic fatty liver disease, nonalcoholic steatohepatitis and liver fibrosis^11–14^. Functional studies of rs1260326 have shown that the minor T allele of a non-synonymous P446L substitution of *GCKR* leads to a weak GCK binding at low glucose concentrations, and impaired response to fructose-6 phosphate^15,16^. We recently demonstrated that the intronic locus including rs780094 is a transcriptional enhancer that regulates *GCKR* expression in a haplotype specific manner^17^.

The important role of GKRP and GCK in the regulation of glucose metabolism in the liver suggests that any functional variant affecting *GCKR*/GKRP could have an effect on glucose metabolism. Lactate is produced by the reduction of pyruvate coupled to the oxidation of reduced nicotinamide adenine dinucleotide (NADH) to NAD^+^. Glucose, contributing to about 65% of circulating lactate^18^ and alanine are the main sources of lactate formation in humans. Plasma lactate levels represent a balance between its formation and clearance. In the fasting state, circulating lactate released mainly from skeletal muscle and adipose tissue is taken up by the liver and used for gluconeogenesis, whereas glucose is metabolized to lactate at high glucose availability^19^. Therefore, blood lactate levels reflect the rate of hepatic glucose metabolism. Previous studies have shown that P446L of *GCKR* is associated with lactate levels in the fasting state^6,10,20^, and after an intake of glucose and fructose^10^.

The aims of our study were, i) to investigate the association of rs780094 of *GCKR* with lactate levels in the fasting state and during hyperglycemia, and ii) to examine the effects of increasing GKRP levels on lactate production to understand the mechanisms underlying the association of rs780094 of *GCKR* with lactate levels. Our study, including human data and overexpression models in HepG2 cells and human primary hepatocytes (HPH), provides new insights into the mechanisms by which rs780094 regulates glucose metabolism in the liver.

## Results

### Association of rs780094 of *GCKR* with lactate levels

**Figure 1:**
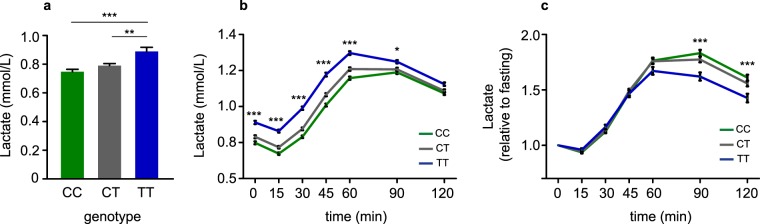
Plasma lactate levels during OGTT. (**a**) Fasting plasma lactate levels according to the rs780094 genotypes of *GCKR* (N = 1,288). Error bars represent the mean ± standard error of the mean (SEM). (**b**) Plasma lactate levels during a seven time point oral glucose tolerance test. (**c**) Plasma lactate levels relative to fasting lactate (*p < 0.05; **p < 0.01; ***p < 0.001, one-way ANOVA).

To investigate the association between the genotypes of rs780094 and lactate levels we performed a seven time point oral glucose tolerance test (OGTT) in 1,288 participants of the METSIM study^8^ (Table 1). Compared to the T allele, the C allele of rs780094 was associated significantly with lower lactate level in the fasting state (p = 5.0 × 10^−6^), at 15 min (p = 5.2 × 10^−9^), 30 min (p = 1.2 × 10^−11^), 45 min (p = 6.3 × 10^−10^), 60 min (p = 5.2 × 10^−7^), and 90 min (p = 0.034) (Fig. 1a,b; Supplementary Table S1). In contrast, the C allele of rs780094 was associated with higher increment in lactate levels relative to fasting lactate levels than the T allele of rs780094 at 90 min (p = 0.0006, after adjustment for insulin level p = 0.0003), and at 120 min (p = 0.0003, after adjustment for insulin level p = 0.0001) (Fig. 1c; Supplementary Table S1), whereas glucose and insulin levels did not differ significantly at any time point (Table 1; Supplementary Table S1). The associations of rs780094 and rs1260326 with lactate levels (fasting and relative to fasting) were very similar in our study, explained by high LD (0.91) between these genetic variants (Supplementary Tables S1, S2).

**Table 1:** Association of the genotypes of rs780094 of the *GCKR* gene with clinical and laboratory measurements in the METSIM study (N = 1,288).

VARIABLE	N	CC (N = 482)	CT (N = 613)	TT (N = 193)	Overall P	p (CC vs TT)
Age (years)	1288	61.5 ± 5.5	61.8 ± 5.4	61.8 ± 5.5	0.767	0.615
Body mass index (kg/m^2^)	1288	27.7 ± 3.7	27.9 ± 3.9	28.2 ± 4.0	0.284	0.128
Waist (cm)	1288	100.2 ± 10.3	100.8 ± 10.4	101.6 ± 11.3	0.276	0.122
Fasting plasma glucose (mmol/l)	1288	5.80 ± 0.49	5.77 ± 0.49	5.7 ± 0.50	0.782	0.525
120 min plasma glucose (mmol/l)	1288	6.00 ± 2.00	5.95 ± 1.91	6.19 ± 2.0	0.406	0.263
Fasting plasma insulin (mU/l)	1288	9.6 ± 6.0	9.7 ± 6.4	9.45 ± 6.17	0.803	0.507
120 min plasma insulin (mU/l)	1286	50.6 ± 49.4	50.6 ± 51.2	54.3 ± 57.4	0.819	0.543
Fasting plasma lactate (mmol/l)	1287	0.75 ± 0.36	0.79 ± 0.37	0.89 ± 0.41	5.0 × 10^−6^	7.2 × 10^−7^
120 min plasma lactate (mmol/l)	1285	1.09 ± 0.38	1.11 ± 0.40	1.16 ± 0.42	0.150	0.051

Mean ± SD, p values based on ANOVA. All p values were obtained from log-transformed variables except for age.

### Effect of increased amounts of GKRP on lactate levels in HepG2 and HPH cells

**Figure 2:**
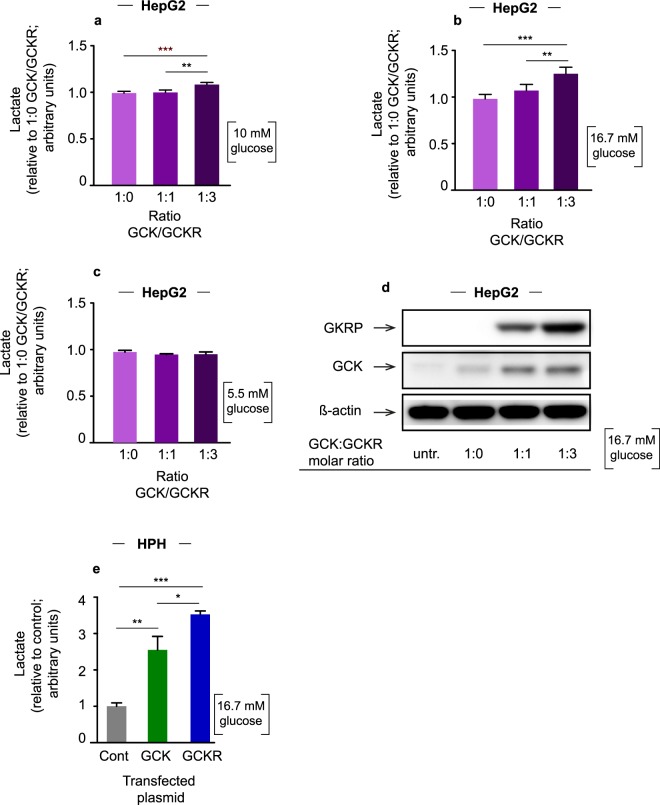
Lactate levels and western blot. (**a**) Lactate levels in HepG2 cells cotransfected with plasmid expressing *GCK* and *GCKR* in three different DNA molar ratios (1:0, 1:1 and 1:3: GCK:GCKR) and stimulated with 10 mM of glucose. (**b**) Cells stimulated with 16.7 mM of glucose. (**c**) Cells stimulated with 5 mM of glucose. The values are presented as relative to 1:0. Error bars represent the standard deviation (SD) (**p < 0.01; ***p < 0.001 one-way ANOVA). (**d**) Cropped images of the representative western blots (at 16.7 mM glucose). Cell extracts from the lactate experiments were used for GCK and GKRP western blots with ß-actin as loading control. Full-length blots are presented in Supplementary Information (Supplementary Fig. S2). (**e**) Lactate levels in HPH cells transfected with plasmid expressing *GCK*, *GCKR* or the control plasmid pCMV6-XL4 and treated with 16.7 mM of glucose. Error bars represent SD (*p < 0.05, **p < 0.01; ***p < 0.001; one-way ANOVA; single donor; 4 technical replicates).

We recently demonstrated that a locus represented by rs780094 is a transcriptional enhancer that regulates *GCKR* expression^17^. We showed that the CGG haplotype, formed by the rs780094, rs780095 and rs780096 genetic variants, had higher transcriptional activity compared to the TAC haplotype, suggesting that differences in *GCKR* expression between the carriers of these two haplotypes may explain previously reported associations of this locus with metabolic parameters, including lactate. To investigate the mechanisms underlying the association of the alleles of rs780094 with lactate levels at different glucose concentrations we cotransfected HepG2 cells with plasmids expressing *GCK* and *GCKR* at three different molar ratios (*GCK*:*GCKR* 1:0, 1:1, 1:3). We found that upon stimulation with high glucose, lactate levels increased depending on the amount of *GCKR* transfected. At 10 mM of glucose, lactate levels increased by 9% (*GCK:GCKR* ratio of 1:3 compared to 1:0, p = 0.001; and *GCK*:*GCKR* ratio 1:1 compared to 1.0, p = 0.002) (Fig. 2a), whereas at 16.7 mM of glucose the increments were 27% (p = 1.3 × 10^−4^) and 16% (p = 0.004), respectively (Fig. 2b). No differences in lactate levels were observed at nondiabetic level of glucose (5.5 mM) (Fig. 2c). Lactate levels did not differ when the cells were cotransfected with *GCK* and the control plasmid pCMV6-XL4, and treated with 16.7 mM of glucose (Supplementary Fig. S1). These results suggest that an increase in lactate levels is dependent on an increase in GKRP levels. We also showed that the levels of GCK increased with GKRP even when the amount of *GCK* plasmid transfected was constant, in agreement with previous studies^21^ (Fig. 2d; Supplementary Fig. S2).

Next we transfected HPH cells from a single male donor with a control plasmid or plasmids expressing *GCK* or *GCKR* and treated these cells with 16.7 mM of glucose. Overexpression of GCK resulted in an increase in lactate levels compared to control (2.5 fold; p = 0.002) (Fig. 2e). Overexpression of GKRP resulted in the highest increase in lactate levels upon glucose stimulation, in agreement with the results in HepG2 cells (3.7-fold compared to control, p = 6.5 × 10^−5^; 1.5-fold compared to GCK overexpression, p = 0.03) (Fig. 2d).

### Effect of glucagon and FOXA2 on *GCKR* expression

**Figure 3:**
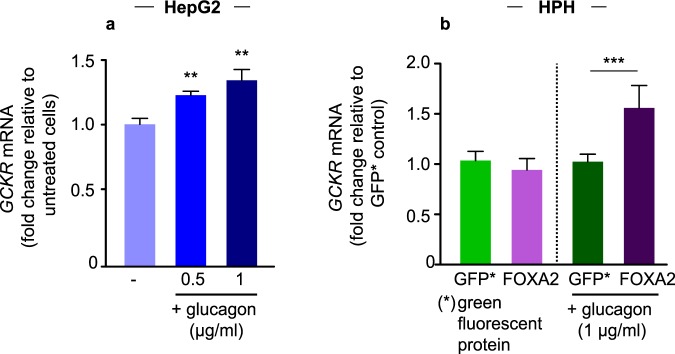
Effect of glucagon on *GCKR* expression in HepG2 and HPH cells. Total *GCKR* mRNA levels were determined using a *GCKR* Taqman Gene Expression Assay. (**a**) HPH cells were serum starved overnight and incubated for 2 h with trichostatin A (TSA; 5 *µ*M) and nicotinamide (Nam; 5 mM) before stimulation with glucagon for 2 h (0.5–1 *µ*g/ml). Error bars represent SD (**p < 0.01; one-way ANOVA; 4 technical replicates with cells from a single donor). (**b**) HepG2 cells were transfected with a plasmid expressing GFP (green fluorescent protein) or FOXA2 as indicated. Cells were treated with glucagon for 6 hours. The results are expressed as relative value to the control GFP. Error bars represent SD (***p < 0.001 one-way ANOVA).

Our previous characterization of an intronic locus including rs780094 demonstrated that a transcriptional enhancer regulates *GCKR* expression in response to FOXA2 in a haplotype specific way (CGG > TAC; rs780094-rs780095-rs780096)^17^. This suggests that FOXA2 activating signals may induce differential *GCKR* transcription through the allele-specific activation of the enhancer by FOXA2. *GCKR* expression is induced by insulin and elevated glucose level in the fed state^22^, whereas FOXA2 is activated by glucagon in the fasting state^23^. Therefore, we investigated the relationship between glucagon and FOXA2 in the activation of *GCKR*. To determine the effect of glucagon on *GCKR* expression we treated HPH cells with two different concentrations of glucagon (0.5 and 1 µg/ml). Our results showed that 0.5 and 1 µg/ml of glucagon increased total *GCKR* expression by 21% (p = 0.03) and 38% (p = 0.01) in these cells (Fig. 3a). To investigate whether the effect of glucagon on *GCKR* expression is dependent on FOXA2 we used HepG2 cells, given its significantly higher transfection efficiency compared to HPH cells. We demonstrated that only the cells transfected with FOXA2 and treated with glucagon showed increased *GCKR* expression compared to control (Fig. 3b). Treatment with a histone acetyl transferase (HAT) inhibitor II reduced *GCKR* expression in FOXA2 transfected, glucagon treated cells, in agreement with a previous study showing that glucagon induces the activation of FOXA2 through acetylation^23^ (Supplementary Fig. S3). Together these results suggest that FOXA2 mediates the effect of glucagon on *GCKR* expression.

## Discussion

In the present study we first investigated the association of rs780094 of *GCKR* with lactate levels in the METSIM study cohort^8,24^. We found that the C allele of rs780094 was associated with decreased lactate levels in the fasting state, but increased lactate levels relative to fasting lactate at 90 and 120 min in an OGTT. Secondly, we showed that GCK amounts and lactate levels increased with increasing GKRP *in vitro* in liver cells, and that the expression of *GCKR* was induced by glucagon.

Our finding that the C allele of rs780094 was associated with higher lactate levels relative to the fasting lactate suggests higher lactate formation upon hyperglycemia. This association was independent of insulin level, indicating that the differences in lactate formation between the carriers of C and T alleles were not explained by the effects of insulin. Our results suggest that decreased lactate levels in the carriers of the C allele in the fasting state indicate increased lactate uptake and higher rate of gluconeogenesis, whereas higher lactate levels upon hyperglycemia indicate higher rates of glycolysis^25–28^.

A previous study in GCKR knockout mice^29^ showed that lactate levels were similar in wild-type and mutant mice in the fasting and fed state. These results, compared to our findings, may be explained by a stronger GCK inhibitory capacity of human GKRP compared with murine GKRP^16,30^. Our results showed that GKRP interaction is crucial for the stabilization and protection of GCK, in agreement with previous studies^21,29,31^. Thus, increased *GCKR*/GKRP expression is expected to increase the formation of a nuclear pool of GKRP-bound GCK, resulting in the translocation of higher amounts of GCK to the cytoplasm at high glucose. Overall, our results suggest that increased lactate levels relative to fasting lactate in carriers of the C allele of rs780094 upon hyperglycemia may be explained by higher *GCKR* expression. However, a limitation of our study is that cellular models using HepG2 cells may not reflect the complexity of hepatic lactate regulation in humans.

Our gene expression data in HPH and HepG2 cells demonstrated that glucagon induced *GCKR* expression *via* FOXA2. However, *GCKR* transcription is induced by insulin and high glucose, a condition equivalent to the fed state^22,32^. While the fed-induced expression of *GCKR* prevents the intracellular accumulation of harmful glucose-6 phosphate and other phosphorylated products^22^, *GCKR* transcription and GKRP expression in the fasting state allows a maximum equilibration and recovery of GCK into the nucleus in conditions where glycolysis is replaced by gluconeogenesis. Thus, our results suggest that FOXA2-dependent mechanism controls *GCKR* expression that may involve an allele-specific activation of rs780094 in the fasting state. Because our results were obtained in HepG2 cells, which are intrinsically mutagenic, and in HPH cells from a single donor, future studies using for example *in vivo* animal models are needed to confirm the effect of fasting on *GCKR* expression.

**Figure 4:**
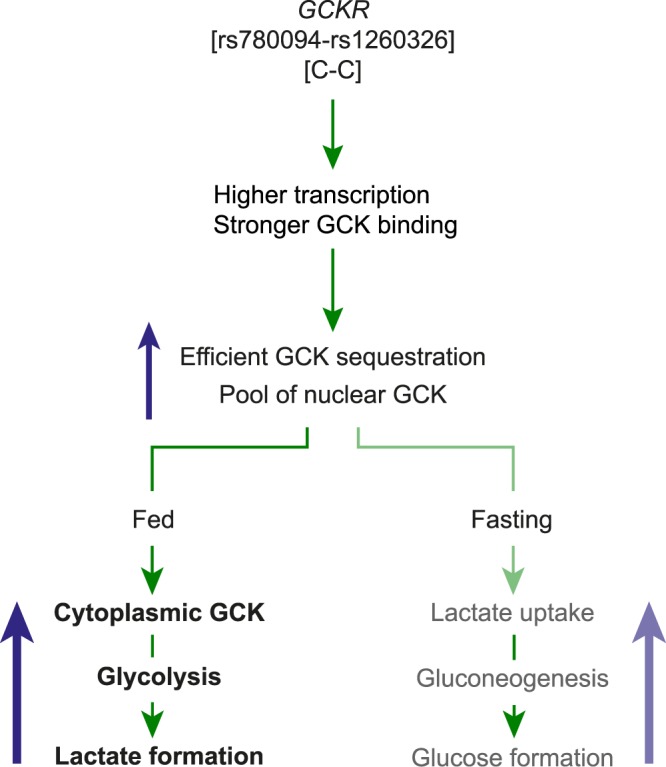
Proposed model for the effects of the C allele of rs780094 on glucose and lactate metabolism in the liver. The characterization of the functional variants of rs780094 and rs1260326 of *GCKR* suggest that the CC haplotype results in more *GCKR* and stronger GKRP-GCK binding, favoring the formation of a richer nuclear pool of the enzyme in the fasting state. Consequently, during hyperglycemia, a richer pool of nuclear GCK would favor more efficient translocation and higher amounts of GCK in the cytoplasm, inducing a higher rate of glycolysis and lactate secretion. In the fasting state, the inhibitory effect of GCK on gluconeogenesis suggests that a more efficient nuclear sequestration of GCK may result in a higher rate of gluconeogenesis from precursors such as lactate, which would increase lactate uptake and glucose formation by the liver.

In addition to rs780094, another functional genetic variant rs1260326 of *GCKR* having a high LD with rs780094, encodes P446L that has a weak binding to GCK at low glucose levels^16^. This may generate a cytoplasmic pool of free GCK that is suggested to induce elevated rate of glycolysis in the fasting state. Thus, our current results, together with our previous work^17^ and the characterization of the P446L variant^16^, allow us to propose a model for the combined effect of these two genetic variants on hepatic glucose metabolism (Fig. 4). The CC haplotype, formed by rs780094 and rs1260326, favors the formation of a richer pool of GCK in the nucleus of the hepatocytes, attributable to higher *GCKR* expression (rs780094-C) and stronger GKRP-GCK binding (rs1260326-C). The CC haplotype favors a higher translocation and activity of GCK in the cytoplasm, resulting in a higher rate of glycolysis and lactate formation (Fig. 4). Since GCK inhibits gluconeogenesis^33^, its efficient nuclear sequestration during fasting favors a higher rate of gluconeogenesis and increased lactate uptake, which results in reduced circulating lactate and increased glucose levels. Our results are in agreement with the notion that targeted molecular inhibition of GKRP could be a promising option in the treatment of type 2 diabetes^34^. Blocking GKRP by GCK-GKRP disruptors promotes GCK cytosolic translocation, and produces antidiabetic effects in rodent models of diabetes^35^.

The strength of our study is that we investigated the associations of lactate levels with *GCKR* variants in an oral glucose tolerance test having multiple measurement points. The limitation of our study is that our subsample of the METSIM cohort is of a moderate size, and included only middle-aged and elderly Finnish men, and therefore we cannot ensure the applicability of our results to women or other populations. Another limitation is that HepG2 cell line shows altered metabolic activity compared to primary hepatocytes, and therefore may not reflect the complexity of hepatic lactate regulation in humans.

In conclusion, our study showed that compared to the carriers of the T allele, the carriers of the C allele of rs780094 of *GCKR* had lower lactate levels in the fasting state but higher lactate increment relative to fasting lactate during hyperglycemia in humans. These results are consistent with a higher hepatic lactate uptake in the fasting state and increased rate of hepatic glycolysis in the fed state. Our findings based on cellular models suggest that increased *GCKR* expression in the fasting state, when accompanied by increased GKRP levels, results in increased rate of hepatic glycolysis in the fed state, as reflected by increased lactate levels.

## Methods

### METSIM study population

The Metabolic Syndrome in Men (METSIM) study is a population-based study including 10,197 Finnish men examined in 2005–2010^8,24^. A total of 1,288 consecutively selected non-diabetic men participating in the METSIM follow-up study were included in the present study. The participants did not differ according to the genotypes of rs780094 with respect to age, BMI or waist (Table 1). The genotyping of rs780094 of *GCKR* was performed using TaqMan Allelic Discrimination Assay at the University of Eastern Finland, as previously described^5^.

### Oral glucose tolerance test and metabolic measurements

An OGTT (75 g glucose) including seven time-points (0, 15, 30, 45, 60, 90 and 120 min) was performed. We calculated a relative increase in lactate levels compared to the fasting lactate. Plasma glucose was measured by enzymatic hexokinase photometric assay (Konelab Systems Reagents, Thermo Fischer Scientific). Insulin was determined using an immunoassay (ADVIA Centaur Insulin IRI, no 02230141, Siemens Medical Solutions Diagnostics), and plasma lactate levels using an enzymatic colorimetric test (Thermo Fischer Scientific).

### Cell culture

HepG2 cells (ATCC, HB-8065) were cultured in Dulbecco’s modified Eagle medium (DMEM; 4.5 g/L glucose, 2 mM L-glutamine, 100 U/ml penicillin, 100 μg/ml streptomycin; LONZA) supplemented with 10% fetal bovine serum (FBS; GIBCO). Cells were seeded at 0.2–0.3 × 10^6^ cells/ml. Cryopreserved HPH cells from a single non-diabetic donor were purchased from Biopredic International and cultured on collagen I coated 24-well plates at 0.8 × 10^6^ cells/ml following the recommended protocol^36,37^. Both HepG2 and HPH cells were incubated on 24 well plates at 37 °C in 5% CO_2_ in a humidified incubator overnight before further manipulations.

### Lactate assay in HepG2 and HPH cells

HepG2 cells were cotransfected with plasmids expressing human GCK (SC127236, Origene) and GKRP (SC119244, Origene) or GCK and the control vector pCMV6-XL4 (Origene) in 1:0, 1:1 and 1:3 plasmid molar ratios (*GCK* to *GCKR*; *GCK* to pCMV6-XL4). All transfections were carried out with Lipofectamine 3000 according to manufacturer’s instructions (Thermo Fisher Scientific). Transfection conditions have been previously optimized in our laboratory. In all cases, we transfected 500 ng of DNA with 0.75 µl of Lipofectamine 3000 reagent per well. Forty-eight hours after transfection the cells were serum starved overnight in low glucose DMEM (1 g/L glucose, 2 mM L-glutamine, 100 U/ml penicillin, 100 μg/ml streptomycin) followed by stimulation with glucose at the indicated concentrations in DMEM (supplemented only with 2 mM L-glutamine). The medium was collected three hours after stimulation for lactate measurement, which was carried out with a colorimetric L-lactate assay according to the manufacturer´s instructions (ab65331, Abcam). The absorbance was measured in a microplate reader at OD 450 nm. Three independent experiments were performed using different cell and DNA preparations. Three to four technical replicates were used for each treatment within each experiment.

HPH cells were transfected with the control vector, or the vectors expressing GCK or GKRP using Tergefect-Hepatocyte transfection kit (Tergeting Systems) according to manufacturer’s instructions. The cells expressed the constructs for 72 hours before conducting the stimulation with glucose and lactate assay as described for HepG2 cells. Three technical replicates per treatment were used.

### Protein immunodetection by Western-blot

HepG2 cells were washed twice with ice cold Phosphate Buffered Saline (PBS) and lysed with radioimmunoprecipitation assay buffer (RIPA; Thermo Fisher Scientific) supplemented with protease inhibitor and phosphatase inhibitor cocktails (Roche). The concentration of proteins was measured with BCA protein assay (Pierce) and 10–15 ug of protein per sample was used in the assay. Samples were prepared with NuPage LDS sample buffer (Life Technologies) and loaded into 4–12% NuPAGE Bis-Tris gels (Life Technologies) for gel electrophoresis. We used the Dual-Color Precision Plus Protein Standards as molecular weight marker (Biorad). Gel-immobilized proteins were then transferred to polyvinylidene fluoride (PVDF) membranes (GE Healthcare) for immunodetection. A mouse monoclonal antibody (RRID:AB_2107650; sc74552, Santa Cruz) and a rabbit polyclonal antibody (RRID:AB_2232078; ab 37796, Abcam) were used for GKRP and GCK detection, respectively. ß-actin was used as loading control and it was detected with a goat polyclonal antibody (RRID:AB_630836; sc1616-R, Santa Cruz). Anti-mouse (NA931V, GE Health care), anti-rabbit (NA934V, GE Health Care) and anti-goat (sc-2020, Santa Cruz) HRP-conjugated IgGs were used accordingly for the detection of the bands by chemiluminiscence (ECL Plus, Pierce). For image acquisition, an Image Quant RT-ECL equipment (GE Healthcare) was used. Band quantitation was carried out with the Gels tool from ImageJ. The list of the antibodies used in this study can be found in Supplementary Table S3.

### Stimulation of HepG2 and HPH cells with glucagon

HepG2 cells were transfected with a plasmid expressing a green fluorescence protein (GFP; PS100040, Origene) or a plasmid expressing human FOXA2 (SC122913, Origene) with Lipofectamine 3000 (Thermo Fisher Scientific) as described above. GFP was used as over-expression control. All constructs were expressed for 48 h before overnight serum starvation. Next, the medium was changed to low glucose serum-free DMEM supplemented with 2 mM L-glutamine and 1 *µ*g/ml of glucagon and incubated in cell culture conditions for 6 h. Since glucagon induces FOXA2 activation through acetylation, we used a HAT inhibitor II (abcam) as a control treatment for 2 h. For stimulation of HPH, we adapted a protocol previously reported to evaluate the effect of glucagon on FOXA2 activation^23^. Briefly, cells were incubated in serum and antibiotics free medium overnight, followed by incubation with the histone deacetylase inhibitors trichostatin A (TSA; 5 µM) and nicotinamide (Nam; 5 mM) for 2 h. Glucagon (0.5 or 1 *µ*g/ml) was added to the medium for 2 h.

### RNA isolation, cDNA preparation and *GCKR* mRNA expression

After glucagon stimulation, both HepG2 and HPH cells were subject to total RNA extraction with an RNeasy Mini Kit (Qiagen). The cDNA was prepared with a High Capacity cDNA Reverse Transcription Kit (Applied Biosystems) using random hexamer primers according to manufacturer´s instructions. *GCKR* mRNA and *RPLP0* mRNA (housekeeping gene; MIM 180510) expression were measured with specific TaqMan gene expression assays (Thermo Fisher Scientific) in a 7500 Real-Time PCR System (Applied Biosystems). The relative gene expression was calculated using the *dd*Ct-quantification method.

### Statistical analysis

Statistical analyses were performed using the SPSS version 25 (IBM). All variables were log-transformed except for age to correct for their skewed distribution. ANOVA was used to compare the three genotype groups, and t test for independent samples to compare the two groups. Statistical analyses applied in cellular experiments are described in the corresponding figure legend. p < 0.05 indicates statistical significance.

## Electronic supplementary material

Supplementary information

## Data Availability

All datasets generated during the current study can be found within the manuscript or the Supplementary Information. Other datasets are available from the corresponding author on request.
