# Supplementary material for: Functional Variant in the GCKR Gene Affects Lactate Levels Differentially in the Fasting State and During Hyperglycemia
Source: Sci Rep. 2018 Oct 30;8:15989. doi: 10.1038/s41598-018-34501-9 (PMC6207693; doi:10.1038/s41598-018-34501-9)
Supplement: Supplementary file 1 — Supplementary information [file 41598_2018_34501_MOESM1_ESM.pdf]

Supplementary Information

Functional Variant in the *GCKR* Gene Affects Lactate Levels Differentially in the Fasting State and During Hyperglycemia

Maykel López Rodríguez[1], Lilian Fernandes Silva[1], Jagadish Vangipurapu[1], Shalem Modi[1], Johanna Kuusisto[2], Minna U. Kaikkonen[3], Markku Laakso[1,2,*].

[1]Institute of Clinical Medicine, Internal Medicine, University of Eastern Finland, Kuopio, Finland.

[2]Department of Medicine, Kuopio University Hospital, P.O. Box 100 FI 70029 KYS, Kuopio, Finland.

[3]A.I. Virtanen Institute for Molecular Sciences, Department of Biotechnology and Molecular Medicine, University of Eastern Finland, Kuopio, Finland.

*****Markku Laakso, MD, PhD, Professor

Institute of Clinical Medicine, Internal Medicine

University of Eastern Finland

70210 Kuopio, Finland

email: markku.laakso@uef.fi

phone: +358 40 672 3338

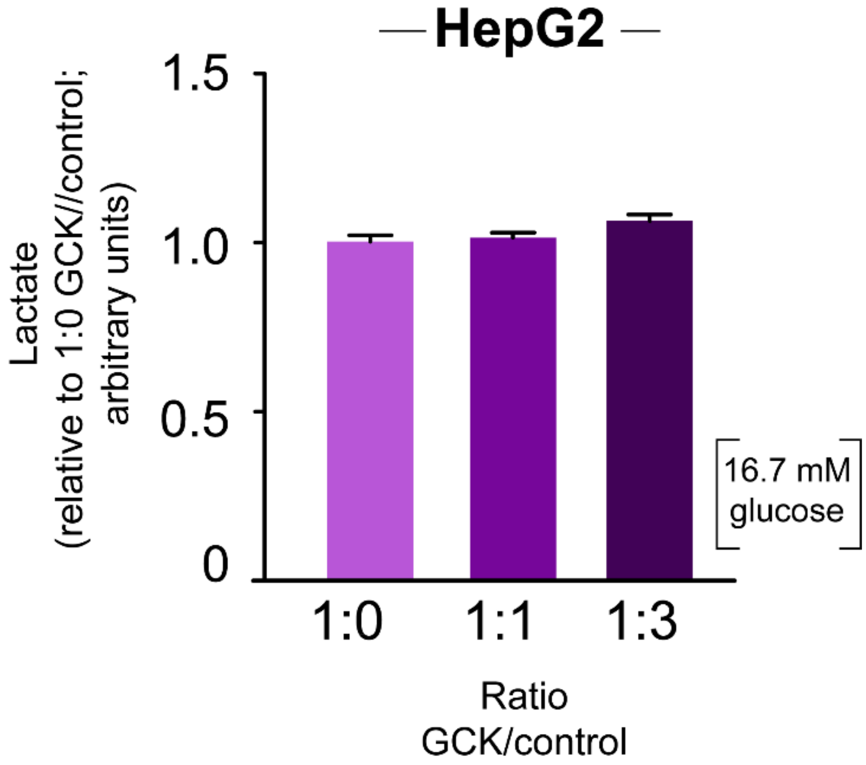

Figure S1. Lactate levels in HepG2 cells. Lactate levels in cells cotransfected with GCK expressing plasmid and control plasmid pCMV6-XL4 and stimulated with 16.7 mM of glucose. The results are presented as relative to 1:0. Error bars represent the standard deviation.

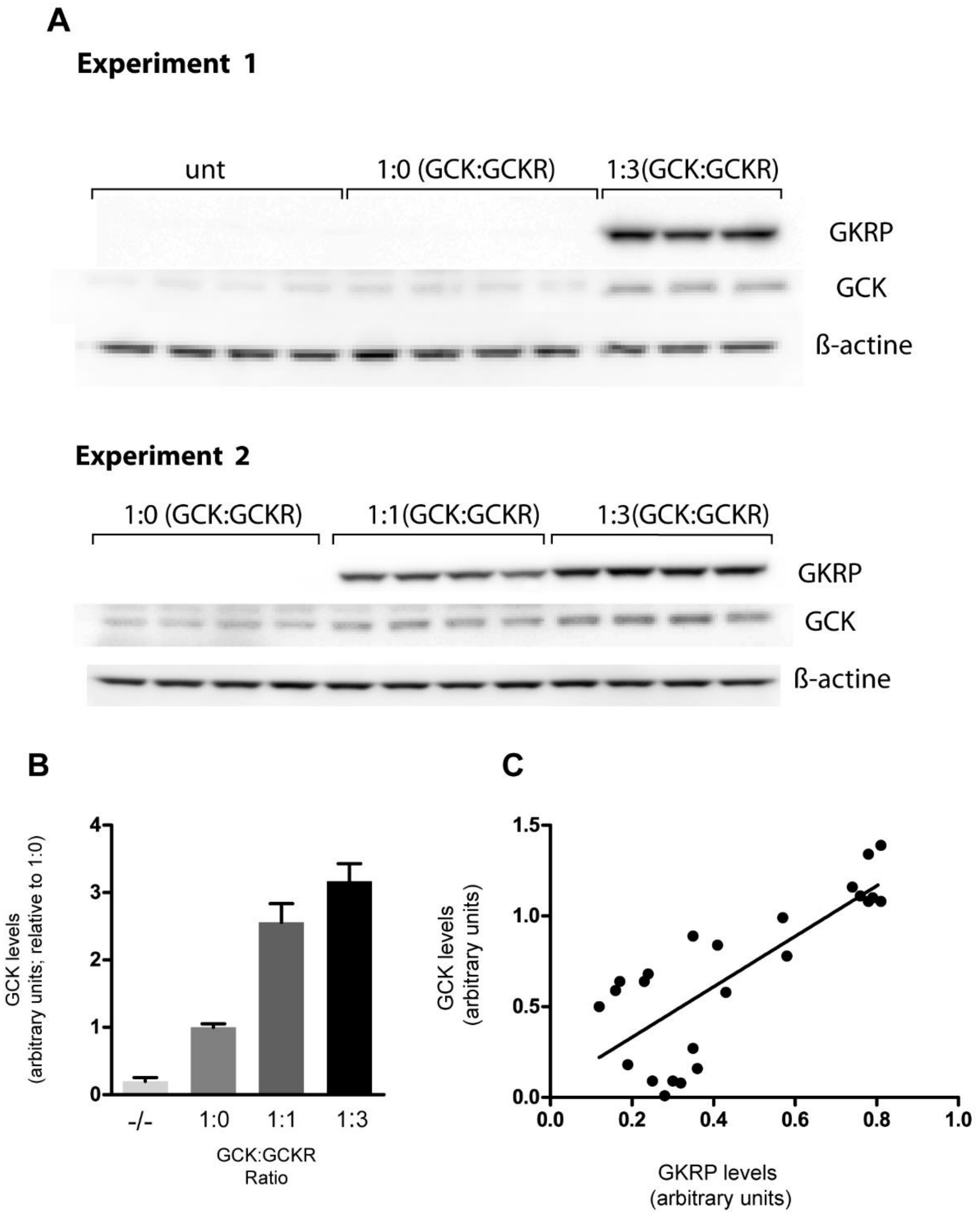

Figure S2. GCK and GKRP Western blot images and quantitation. A Representative western blots (GCK; GKRP; ß-actin) from protein extracts corresponding to the cells used in lactate experiments (cropped images) (Experiments 1 and 2 in Figure 2). The detection

of GKRP, GCK and ß-actin was performed in the same membrane. Full-length blots images are presented after Supplementary Figure 3. B Quantitation of GCK protein bands intensity, (relative values with respect to ratio 1:0). The quantitation includes the cell extracts from 5 independent experiments with ≥ 3 technical replicates each (including the protein preparation from cells used in lactate experiments). GCK levels increase with GKRP. C Scatter plot of band intensity (GCK vs GKRP). Only the pairs of values where GKRP level was detectable were used. GCK and GKRP levels are positive correlated (Spearman´s correlation coefficient 0.774; p=3.8 × 10^{-5}). (full blot images are presented after Fig. S4).

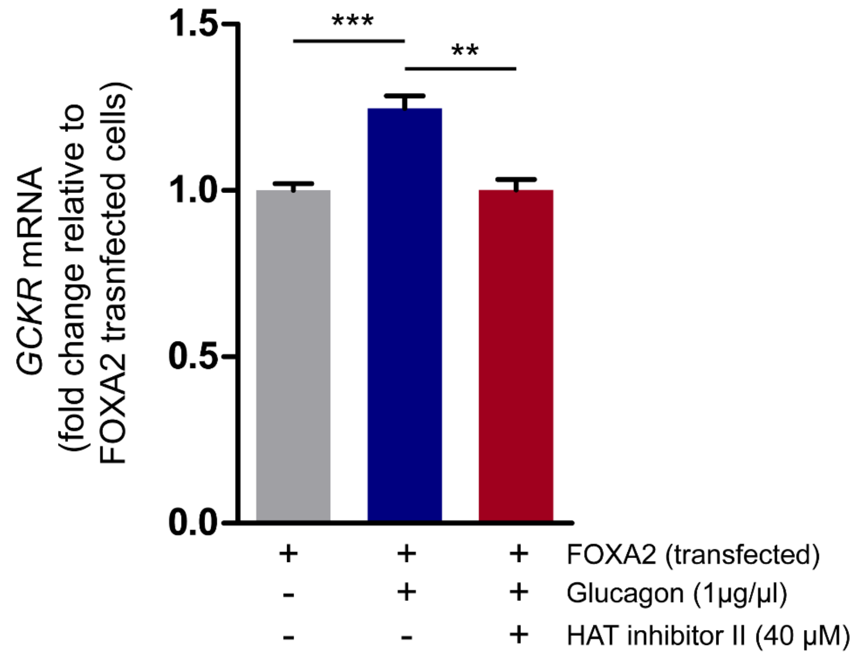

Figure S3. Effect of histone acetyl transferase (HAT) inhibitor II on *GCKR* expression in HepG2 cells. Total *GCKR* mRNA levels were determined using a *GCKR* Taqman Gene Expression Assay. HepG2 cells were transfected with a plasmid expressing FOXA2 and treated with glucagon (6 hours) and HAT inhibitor II (2 hours) as indicated. HAT inhibitor II reduced *GCKR* expression. The results are expressed as relative value to untreated cells. Error bars represent SD (***$p < 0.001$; **$p < 0.005$;two-tailed *t* test).

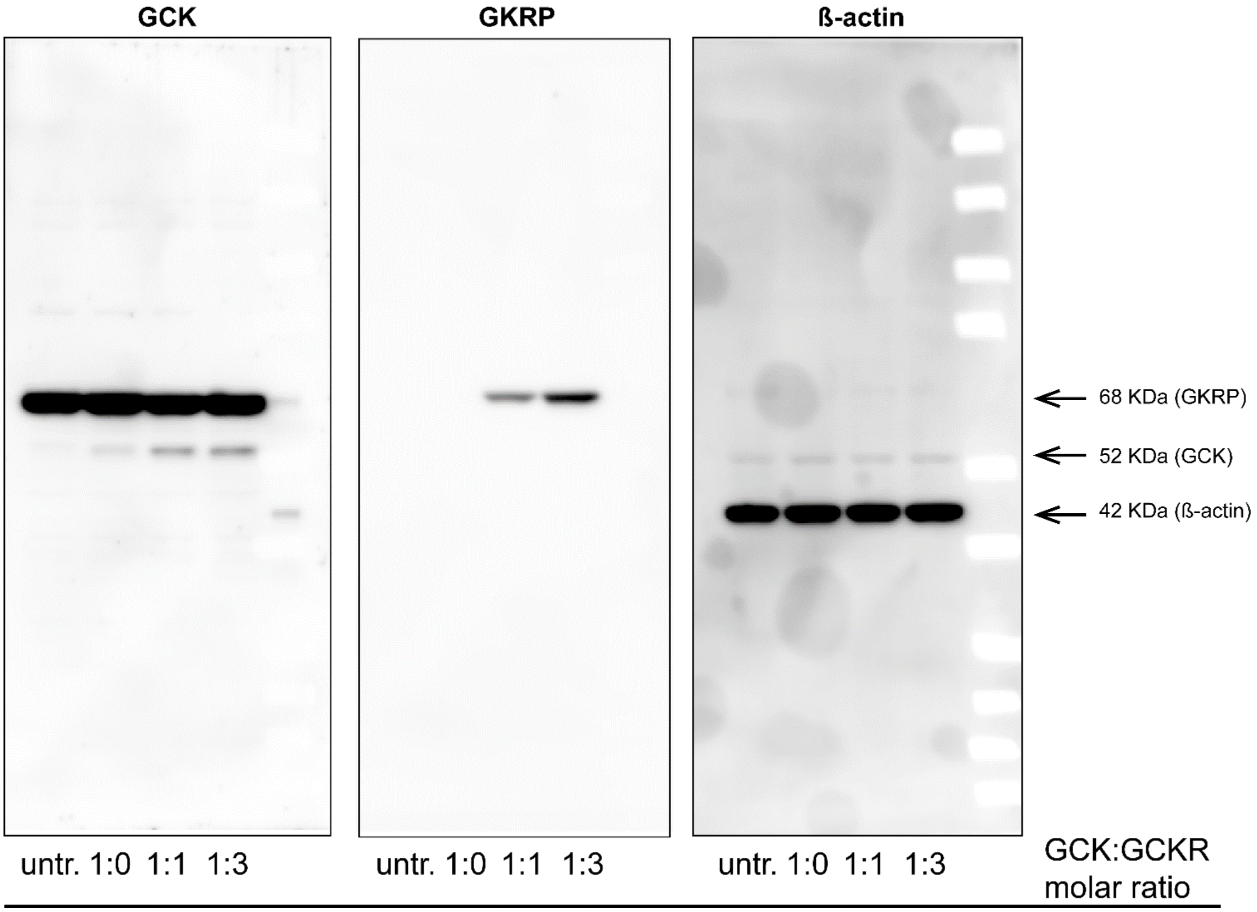

Fig. S4. Full western blot images for main Figure 2D

Full blot images for Supplementary Fig. S2, upper panel (Experiment 1).

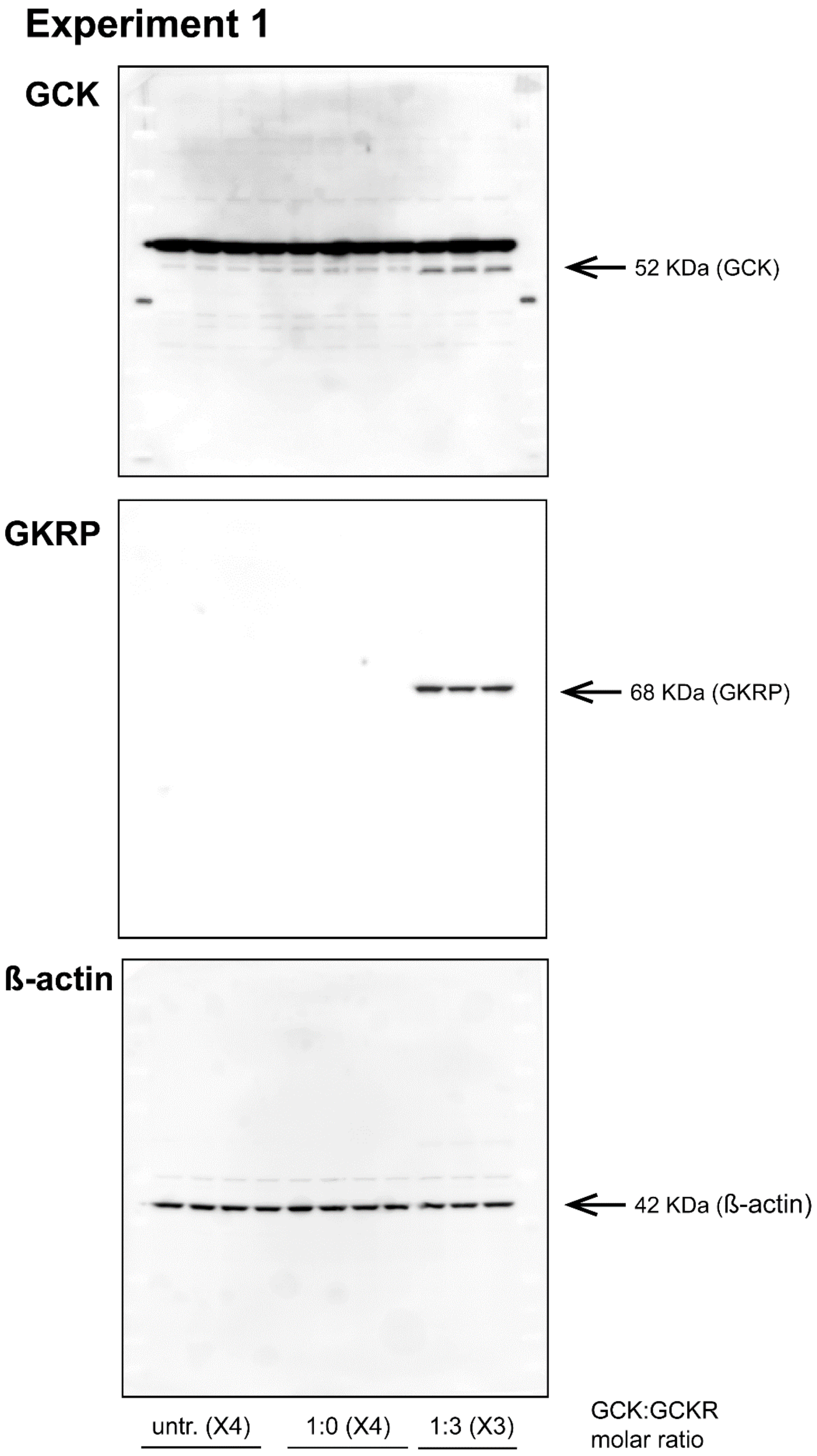

Full blot images for Supplementary Fig. S2, lower panel (Experiment 2).

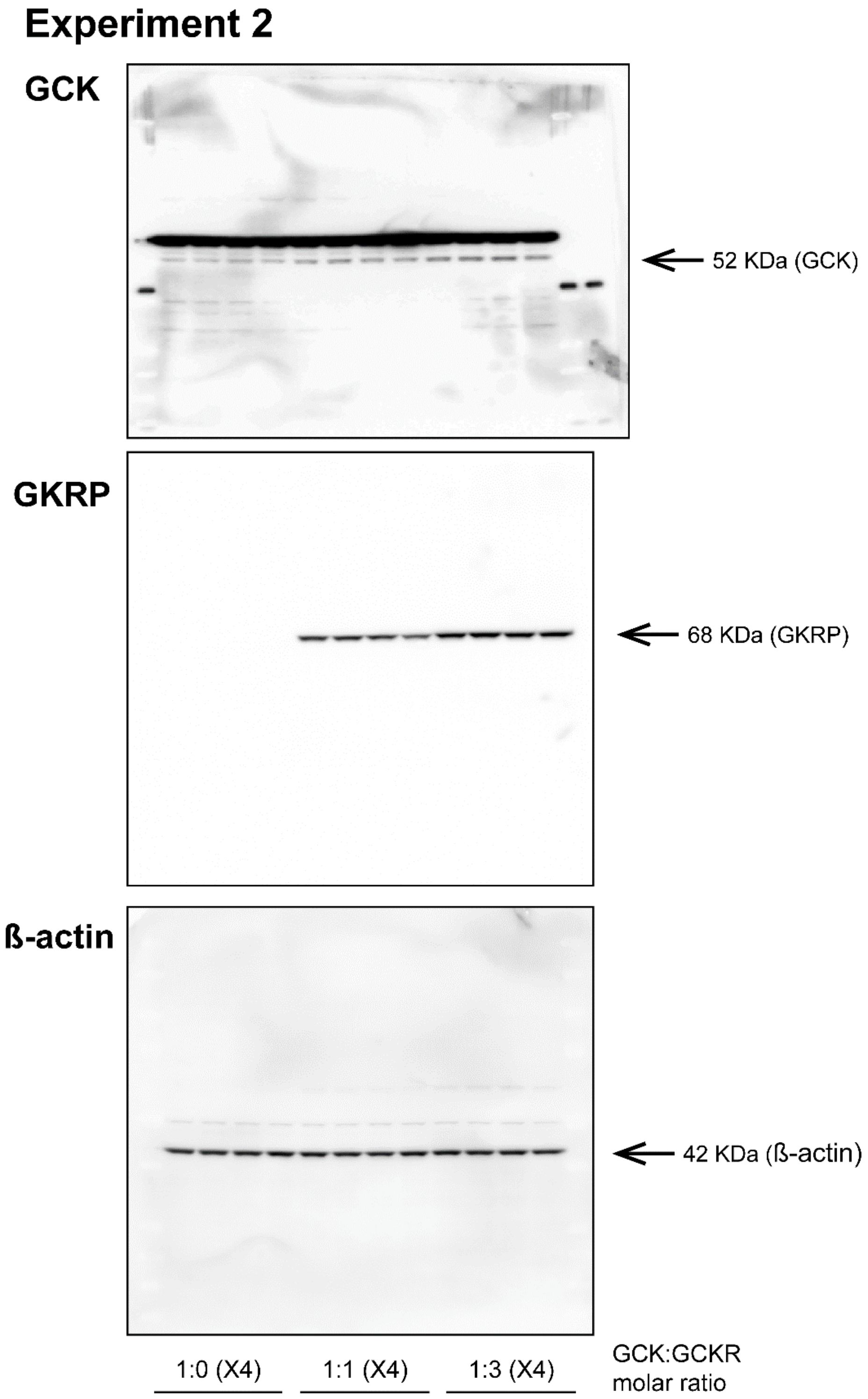

Table S1. Association of lactate, glucose and insulin levels with *GCKR* rs780094 in METSIM study in an oral glucose tolerance test (N=1288)

Lactate (mmol/L)	N	CC (N=482)	CT (N=613)	TT (N=193)	Overall P	CC vs TT P	
0 min	1287	0.75 ± 0.36	0.79 ± 0.37	0.89 ± 0.41	**5.0×10^{-6}**	**7.2×10^{-7}**	
15 min	1286	0.67 ± 0.29	0.72 ± 0.31	0.83 ± 0.36	**5.2×10^{-9}**	**5.8×10^{-10}**	
30 min	1288	0.79 ± 0.31	0.85 ± 0.33	0.99 ± 0.40	**1.2×10^{-11}**	**1.2×10^{-12}**	
45 min	1286	1.01 ± 0.36	1.08 ± 0.39	1.22 ± 0.46	**6.3×10^{-10}**	**2.1×10^{-10}**	
60 min	1286	1.20 ± 0.39	1.26 ± 0.42	1.37 ± 0.45	**5.2×10^{-7}**	**1.2×10^{-7}**	
90 min	1285	1.24 ± 0.41	1.26 ± 0.39	1.31 ± 0.41	**0.034**	**0.0121**	
120 min	1285	1.09 ± 0.38	1.11 ± 0.40	1.16 ± 0.42	0.151	0.0506	
*Normalized Lactate**							**P***
15 min	1286	0.94 ± 0.20	0.94 ± 0.21	0.96 ± 0.19	0.379	0.156	0.177
30 min	1288	1.12 ± 0.31	1.13 ± 0.30	1.16 ± 0.29	0.191	0.073	0.128
45 min	1286	1.47 ± 0.47	1.49 ± 0.49	1.47 ± 0.42	0.746	0.832	0.677
60 min	1286	1.77 ± 0.59	1.76 ± 0.60	1.67 ± 0.47	0.312	0.136	0.255
90 min	1285	1.83 ± 0.64	1.78 ± 0.62	1.62 ± 0.50	**0.0006**	**0.0001**	**0.00033**
120 min	1285	1.61 ± 0.57	1.56 ± 0.62	1.43 ± 0.53	**0.0003**	**4.2×10^{-5}**	**0.00011**
Glucose (mmol/L)							
0 min	1288	5.77 ± 0.49	5.77 ± 0.49	5.75 ± 0.50	0.782	0.525	
15 min	1288	7.50 ± 1.01	7.47 ± 0.99	7.43 ± 1.04	0.723	0.436	
30 min	1286	9.34 ± 1.47	9.26 ± 1.47	9.33 ± 1.42	0.620	0.985	
45 min	1286	9.86 ± 2.02	9.81 ± 2.04	9.92 ± 2.00	0.694	0.609	
60 min	1288	9.56 ± 2.40	9.42 ± 2.44	9.58 ± 2.36	0.466	0.824	
90 min	1288	7.44 ± 2.39	7.45 ± 2.46	7.64 ± 2.47	0.605	0.356	
120 min	1285	6.00 ± 1.97	5.95 ± 1.91	6.19 ± 2.03	0.406	0.263	
Insulin (mU/l)							
0 min	1288	9.65 ± 6.03	9.72 ± 6.42	9.45 ± 6.17	0.803	0.507	
15 min	1286	36.77 ±	37.31 ±	37.57 ±	0.855	0.695	
30 min	1287	64.24 ±	66.64 ±	68.96 ±	0.363	0.165	
45 min	1286	85.87 ±	87.48 ±	90.58 ±	0.312	0.126	
60 min	1285	92.44 ±	91.85 ±	94.69 ±	0.591	0.356	
90 min	1287	78.65 ±	78.74 ±	79.03 ±	0.869	0.650	
120 min	1286	50.64 ±	50.60 ±	54.34 ±	0.819	0.543	

Mean ± SD and P values are based on ANOVA. All P values were obtained from log-transformed variables. *Normalized levels were obtained by dividing raw values at each time point by those at 0 min, respectively. P is unadjusted, P* adjusted for insulin levels at the corresponding time points.

Table S2. Association of lactate, glucose and insulin levels with *GCKR* rs1260326 in METSIM study in an oral glucose tolerance test (N=1288)

Lactate (mmol/L)	N	CC (N=493)	CT (N=593)	TT (N=188)	Overall P	CC vs TT P	
0 min	1287	0.74 ± 0.36	0.80 ± 0.37	0.89 ± 0.40	**3.9×10^{-7}**	**6.4×10^{-8}**	
15 min	1286	0.67 ± 0.29	0.72 ± 0.31	0.83 ± 0.5	**1.9×10^{-9}**	**1.4×10^{-10}**	
30 min	1288	0.79 ± 0.31	0.85 ± 0.34	0.99 ± 0.40	**3.6×10^{-12}**	**2.2×10^{-13}**	
45 min	1286	1.01 ± 0.35	1.09 ± 0.40	1.22 ± 0.45	**2.3×10^{-10}**	**4.6×10^{-11}**	
60 min	1286	1.19 ± 0.39	1.27 ± 0.43	1.37 ± 0.45	**2.0×10^{-7}**	**2.0×10^{-8}**	
90 min	1285	1.24 ± 0.40	1.26 ± 0.40	1.31 ± 0.41	**0.029**	**0.012**	
120 min	1285	1.09 ± 0.38	1.11 ± 0.40	1.15 ± 0.43	0.163	0.059	
*Normalized Lactate**							**P***
15 min	1286	0.94 ± 0.20	0.94 ± 0.21	0.96 ± 0.20	0.585	0.376	0.177
30 min	1288	1.13 ± 0.30	1.13 ± 0.29	1.16 ± 0.29	0.397	0.188	0.128
45 min	1286	1.48 ± 0.47	1.49 ± 0.48	1.46 ± 0.43	0.904	0.803	0.677
60 min	1286	1.78 ± 0.59	1.75 ± 0.59	1.66 ± 0.50	0.157	0.053	0.255
90 min	1285	1.85 ± 0.64	1.77 ± 0.61	1.60 ± 0.49	**0.00006**	**0.00001**	**0.00003**
120 min	1285	1.63 ± 0.57	1.55 ± 0.62	1.41 ± 0.53	**0.00002**	**3.0×10^{-5}**	**0.000007**
Glucose (mmol/L)							
0 min	1275	5.77 ± 0.48	5.77 ± 0.49	5.75 ± 0.50	0.880	0.625	
15 min	1274	7.50 ± 1.01	7.45 ± 0.98	7.43 ± 1.03	0.615	0.387	
30 min	1275	9.35 ± 1.47	9.25 ± 1.45	9.33 ± 1.43	0.555	0.938	
45 min	1274	9.86 ± 2.00	9.80 ± 2.03	9.92 ± 2.04	0.736	0.703	
60 min	1275	9.56 ± 2.38	9.42 ± 2.44	9.57 ± 2.40	0.506	0.863	
90 min	1275	7.43 ± 2.39	7.46 ± 2.47	7.63 ± 2.49	0.649	0.363	
120 min	1275	5.99 ± 1.97	5.97 ± 1.92	6.20 ± 2.02	0.390	0.208	
Insulin (mU/l)							
0 min	1275	9.66 ± 6.12	9.64 ± 6.17	9.57 ± 6.35	0.826	0.536	
15 min	1273	36.91 ± .46	37.07 ± 26.60	37.83 ± 25.44	0.802	0.671	
30 min	1274	64.45 ± 43.06	66.55 ± 45.95	68.88 ± 47.21	0.411	0.195	
45 min	1273	86.03 ± 59.93	86.93 ± 58.47	91.34 ± 57.71	0.251	0.099	
60 min	1273	93.22 ± 66.68	90.64 ± 60.70	95.53 ± 62.42	0.493	0.313	
90 min	1274	78.65 ± 68.63	77.71 ± 65.41	80.16 ± 66.31	0.767	0.504	
120 min	1273	50.70 ± 51.23	50.00 ± 49.57	55.31 ± 58.66	0.706	0.418	

Mean ± SD and P-values based on ANOVA. All P-values were obtained from log-transformed variables.
*Normalized levels were obtained by dividing raw values at each time point by those at 0 min respectively.
P is unadjusted, P* adjusted for insulin levels at corresponding time points.

Table S3. List of antibodies

**Target**	**Antibody cat. number**	**RRID**	**Vendor**
GCK	ab37796	RRID:AB_2107650	Santa Cruz Biotechnology
GKRP	sc74552	RRID:AB_2232078	Abcam
ß-actin	sc1616-R	RRID:AB_630836	Santa Cruz Biotechnology
Mouse IgG	NA931V	-	GE Health care
Goat IgG	sc-2020	RRID:AB_631728	Santa Cruz Biotechnology
Rabbit IgG	NA934V	-	GE Health care

Supplementary Methods.

Western blot for the three proteins in a single experiment were performed on the same membrane. Any cropping to the images was performed with the purpose of presentation only. Brightness and contrast adjustments were performed with ImageJ B&c tool and always applied to the whole image. Quantification was performed on a single membrane. All comparisons refer to samples in the same membrane. Each point of the scatter plot in Fig. S2 represents the GCK vs GKRP value after normalization to the corresponding beta-actin signal for that sample. We used Dual-Color Precision Plus Protein Standards as molecular weight marker (Biorad; cat.#: 161-0364). Visible images were taken before and superimposed to the radio images to verify the size of the expected band.
